# Rare musculoskeletal diseases in adults: a research priority setting partnership with the James Lind Alliance

**DOI:** 10.1186/s13023-020-01398-5

**Published:** 2020-05-19

**Authors:** Gerda Mickute, Kristina Staley, Heather Delaney, Oliver Gardiner, Amy Hunter, Richard Keen, Lorraine Lockhart, Nick Meade, Maria Newman, Stuart Ralston, Elaine Rush, Sheela Upadhyaya, Sandra Regan, Laura Watts, Jennifer Walsh, Paul White, Roger M. Francis, M. Kassim Javaid

**Affiliations:** 1 Somerville College, University of Oxford, Oxford, OX2 6HD UK; 2 Montague House, 4 St. Mary’s Street, Ross on Wye, HR9 5HT UK; 3 Patient representative, FDSS UK, London, UK; 4 Patient representative, XLH Network, London, UK; 5 Genetic Alliance UK, N1 6AH, London, UK; 6 Royal National Orthopaedic Hospital, Stanmore, HA7 4LP UK; 7 Patient, Banbury, Oxfordshire UK; 8 Patient, London, UK; 9 MRC Institute of Genetics and Molecular Medicine, University of Edinburgh, Edinburgh, EH4 2XU UK; 10 Independent Adviser of the James Lind Alliance, London, UK; 11 NIHR Oxford Biomedical Research Centre Project Manager, Oxford, UK; 12 NIHR Musculoskeletal Biomedical Research Unit, University of Oxford, Oxford, OX3 7LD UK; 13 Metabolic Bone Centre, Sheffield Teaching Hospitals NHS Foundation Trust, S5 7AU, Sheffield, UK; 14 Translational and Clinical Research Institute, Newcastle University, Newcastle upon Tyne, NE1 7RU UK; 15 The Botnar Research Centre, Nuffield Department of Orthopaedics, Rheumatology and Musculoskeletal Sciences, University of Oxford, Oxford, OX3 7LD UK

**Keywords:** James Lind Alliance, Priority setting partnership, Fibrous dysplasia/McCune-Albright syndrome, X-linked hypophosphatemia, Osteogenesis imperfecta, Rare musculoskeletal diseases, Research priorities, Patient and public involvement, Research mismatch

## Abstract

**Background:**

Osteogenesis imperfecta, fibrous dysplasia/McCune-Albright syndrome and X-linked hypophosphatemia are three rare musculoskeletal diseases characterised by bone deformities, frequent fractures and pain. Little high-quality research exists on appropriate treatment and long-term management of these conditions in adults. This is further worsened by limited research funding in rare diseases and a general mismatch between the existing research priorities and those of the patients. This partnership adopted the James Lind Alliance approach to identify the top 10 research priorities for rare musculoskeletal diseases in adults through joint patient, carer and healthcare professional collaboration.

**Results:**

The initial survey for question collection recruited 198 respondents, submitting a total of 988 questions. 77% of the respondents were patients with a rare musculoskeletal disease. Following out-of-scope question exclusion, repeating query grouping and scientific literature check for answers, 39 questions on treatment and long-term management remained. In the second public survey, 220 respondents, of whom 85% were patients with a rare musculoskeletal disease, their carers, relatives or friends, prioritised these uncertainties, which allowed selection of the top 25. In the last stage, patients, carers and healthcare professionals gathered for a priority setting workshop to reach a consensus on the final top 10 research priorities. These focus on the uncertainties surrounding appropriate treatment and holistic long-term disease management, highlighting several aspects indirect to abnormal bone metabolism, such as extra-skeletal symptoms, psychological care of both patients and their families and disease course through ageing.

**Conclusions:**

This James Lind Alliance priority setting partnership is the first to investigate rare bone diseases. The priorities identified here were developed jointly by patients, carers and healthcare professionals. We encourage researchers, funding bodies and other stakeholders to use these priorities in guiding future research for those affected by rare musculoskeletal disorders.

## Background

Fibrous dysplasia/McCune-Albright Syndrome (FD/MAS), X-linked hypophosphatemia (XLH) and osteogenesis imperfecta (OI) are rare genetic musculoskeletal disorders characterised by bone pain, lesions and deformities as well as a number of other extra-skeletal symptoms and complications. Limited treatment options are available for these conditions and wide gaps of knowledge persist in natural disease history, long-term risks, disease management and quality of life in those affected. Consequently, there is little awareness amongst healthcare professionals on the diagnosis and management of these disorders, which is further complicated by natural variability in clinical presentation and disease burden [1–3]. Therefore, it may take years for some patients before appropriate diagnosis is given and high-quality specialist care is accessed.

These issues are worsened by limited research funding in rare diseases and the apparent detachment of existing research efforts and knowledge base from patient, carer and healthcare professional research priorities [4, 5]. However, over the last three decades patient involvement in research has been increasingly recognised and valued in terms of providing needs-led research priorities for attaining best patient care [6–8]. The James Lind Alliance (JLA), hosted by the National Institute for Health Research, is a non-profit initiative providing a transparent, rigorous approach supervised by an impartial JLA adviser which brings patients, carers and healthcare professionals together for a research priority setting partnership (PSP) [9, 10]. Since its establishment in 2004, numerous PSPs have been carried out on a range of disorders, further reaffirming marked differences between the priorities of researchers and those identified within the PSPs [11, 12].

Several PSPs have been completed for common musculoskeletal conditions [13–17], however none addressed rare musculoskeletal disorders. This PSP applied the JLA method to a group of rare musculoskeletal disorders in adults – FD/MAS, XLH and OI, in order to identify the most important research directions in diagnosis, treatment and long-term management.

## Methods

**Fig. 1:**
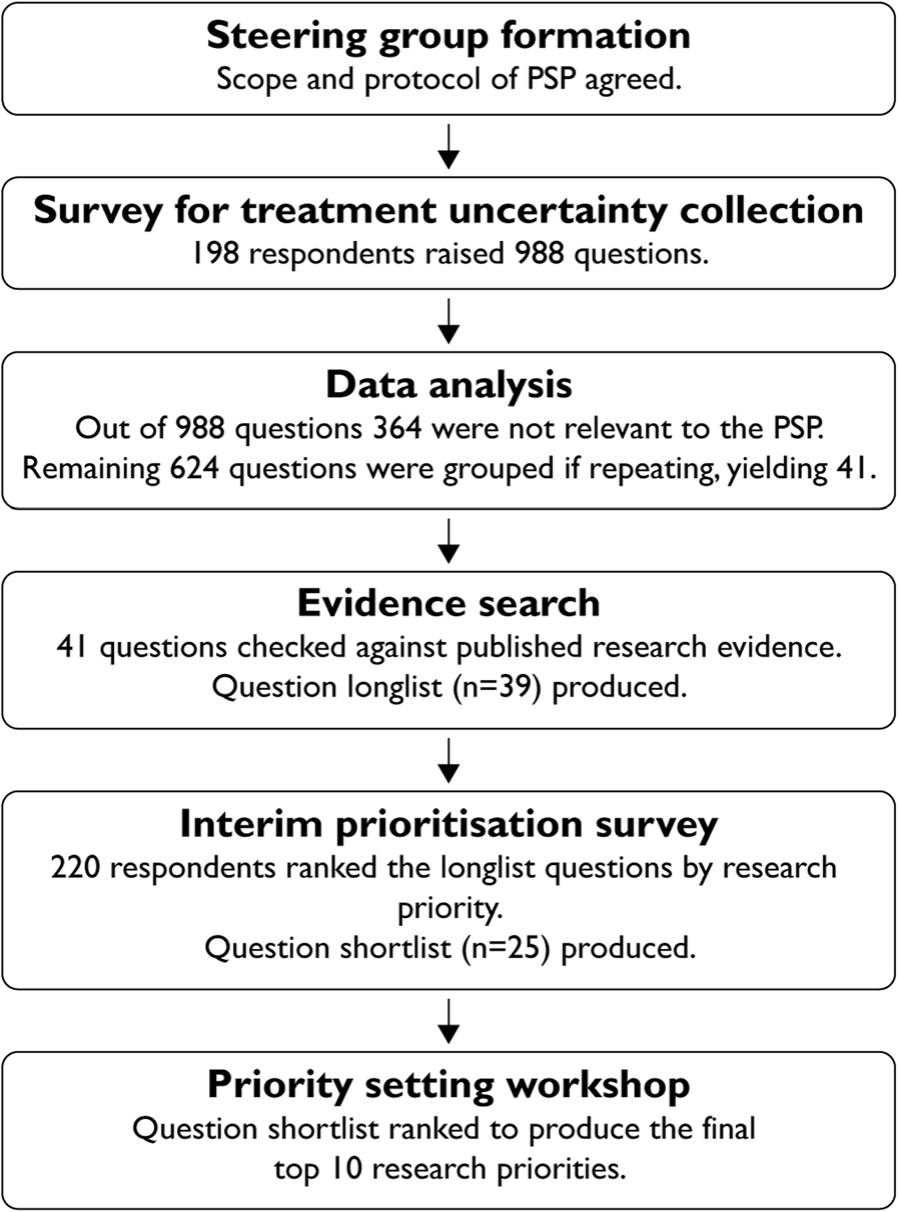
Flowchart summarizing the rare musculoskeletal diseases in adults PSP process

This PSP aimed to identify research uncertainties common to rare musculoskeletal disorders and related to their treatment and long-term management. The rigorous and transparent JLA PSP approach, outlined in Fig. 1, involved patients, carers and healthcare professionals. It was overseen by an impartial JLA adviser and a JLA project manager to ensure equal and fair participant representation. This partnership took place from December 2015 to November 2018.

### Steering group formation

The steering group was assembled to oversee and lead the PSP through its every stage. Fair representation of patients, carers and healthcare professionals was ensured by including both the patient representatives (*n* = 6) and healthcare professionals (*n* = 5). As dictated by the JLA methodology, pure researchers were excluded from the PSP process as their voice is already represented by the majority of the existing scientific literature [10]. The members of the steering group were asked to declare any competing interests.

The steering group committed to publicising the PSP surveys and outcome through their connections to stakeholders and relevant communities. In order to gather appropriate patient and public involvement, the PSP was conducted in collaboration with the following patient support organisations and charities: Brittle Bone Society, The Fibrous Dysplasia Support Society, XLH Network, XLHUK and Genetic Alliance UK. Furthermore, the steering group supervised survey development and data analysis, recruited appropriate individuals to conduct an evidence search and participated in the final priority setting workshop. The steering group ensured that the PSP was inclusive and fair to all the participating groups and representatives, considering both the practical and the methodological aspects of the project.

### Scope

This PSP focused on adult patients with XLH, OI or FD/MAS, their carers and healthcare professionals. These disorders were chosen because of the active patient groups they have. The focus on adults, defined as aged 16 and above, aimed to capture adult patient underrepresentation in the current scientific knowledge base and potential uncertainties arising during patient transition from paediatric to adult services. The scope of this PSP was defined to include uncertainties on treatment, long-term management and diagnosis of the disorders. The latter was recognised as a potentially challenging topic to cover considering limited PSP resources. Thus, it was agreed that the steering group would make most appropriate decisions on the use of such uncertainties depending on the responses received from the survey for question collection. Lastly, the steering group agreed to expand survey distribution to certain other countries with similar standards of care, namely Spain, Italy, France, Netherlands, Germany, Denmark and Norway, in order to maximise the number of responses for these rare disorders. Due to its limited resources, the PSP accepted survey responses applicable only to the UK healthcare system and written in English.

### Survey for question collection

The initial survey was designed to ask patients, their carers and healthcare professionals for any questions they had about one of the three rare disorders. It was composed online using SurveyMonkey and as a PDF for printing and local distribution to those unable to access it online. Before its release, the steering group made sure the survey was comprehensible for all potential respondents, no matter their scientific background. The steering group members, collaborating patient support organisations and charities used their contacts to disseminate the survey via email, social media, newsletters, blogs and websites. The survey collected basic demographic data, including respondent type (patient, carer or clinician), name of the rare musculoskeletal disease the patient has (XLH, FD/MAS or OI), gender and country of residence, in addition to the free text answers for the questions respondents had. The answers could be submitted from March to July 2017.

### Survey response categorisation and evidence search

Submitted questions were assessed for their relevance to the scope of this PSP. They were omitted from the further process if they were off-topic, too broad, unclear or no scientific research was required to answer them. Furthermore, questions specific to a non-UK healthcare system were omitted as well. The remaining uncertainties were grouped into overarching questions if repeated across respondents. This stage of the PSP was performed by an information specialist and was overseen by the steering group to ensure clarity and accurate collation of the questions.

In the next stage, the remaining uncertainties were checked against the existing high-quality scientific evidence, namely systematic reviews, clinical trials and guidelines. Uncertainties that could be reliably answered, i.e. the “unrecognised knowns”, were excluded from the further PSP process. Some questions had limited scientific literature to answer them and additional discussion by the steering group determined whether they were included in the further stages of the PSP. This produced the longlist of questions.

### Interim prioritisation survey

The aim of this survey was to prioritise the longlist of questions and produce a shortlist for the final priority setting workshop. The survey was composed online using SurveyMonkey. It was piloted by the steering group and distributed via the same channels as the first survey. It collected basic demographic data from its respondents (described in “Survey for question collection”) and asked to select the ten most important questions from the presented longlist, where their order was automatically randomised for each participant. Then, the respondent was able to rank the selected questions from the most to least important. The answers could be submitted from April to May 2018.

The survey responses were used to create a prioritised question longlist. In order to do so, four separate rankings were produced according to the type of respondent: 1) FD/MAS, 2) OI, 3) XLH patients and carers as well as 4) healthcare professionals. The rankings were combined to yield an overall ranking position for each question, which ensured equal representation of the four groups in the exercise. The top 25 questions were selected for proceeding to the final priority setting workshop.

### Priority setting workshop

Over the duration of a single day, the priority setting workshop was designed to bring together patients, carers and healthcare professionals in order to produce the top 10 research priorities from the shortlist of questions. The participants (*n* = 18) were members of the steering group and other representatives recruited via previously used dissemination channels. Equal representation of patients, carers and healthcare professionals was ensured. The workshop comprised several small group discussions, where all participants were able to share their views. This process allowed the group to reach a consensus on the final top 10 research uncertainties. The JLA adviser was present during the workshop to mediate the discussion and ensure equal contribution of the participants. To guarantee transparency, those participating in the priority setting workshop were asked to declare any competing interests. The event was held at Friends Meeting House in London in June 2018.

## Results

The summary of the PSP process is presented in Fig. 1. The survey for question collection received 198 responses, gathering a total of 988 questions. Most respondents identified as patients with one of the three rare musculoskeletal disorders (77%), whilst others indicated being carers, relatives or friends (11%), health and social care professionals (11%) or representatives of organisations (1%).

Following the categorisation of survey results, 364 questions out of the 988 were identified as not relevant to this PSP. Out of those, 186 did not require scientific research to be answered, 65 inquired about healthcare professional training and service access and 113 were considered unclear, too broad or off-topic. The remaining questions were grouped into overarching questions if repeated, yielding a total of 41 questions. These were checked against published high-quality research evidence and two questions were identified as “unrecognised knowns”, where sufficient scientific evidence existed to reliably answer them:

1. Could diagnosis in adults be made faster and more accurate e.g. through use of genetic or other biomedical tests?
2. What is the optimal imaging technique for fibrous dysplasia?

The remaining questions (*n* = 39) comprised the PSP longlist. Thematically, questions addressed treatment (*n* = 14), prognosis (*n* = 8), self-management (*n* = 3), support and care (*n* = 3), healthcare (*n* = 3) and prevention (*n* = 1). Several other questions did not adhere to any of these thematic groups (*n* = 7).

The longlist was prioritised via the interim prioritisation survey, where 220 submissions were received. Most of the respondents were patients, carers, relatives or friends (85%), whilst others were healthcare professionals (14%) and representatives of organisations (1%). Most of the respondents were female (83%) and from outside of the UK (56%). The respondent ethnic group was white by a large majority (94%). This prioritisation exercise allowed the steering group to shortlist the top 25 research uncertainties for the final stage of the PSP.

**Fig. 2:**
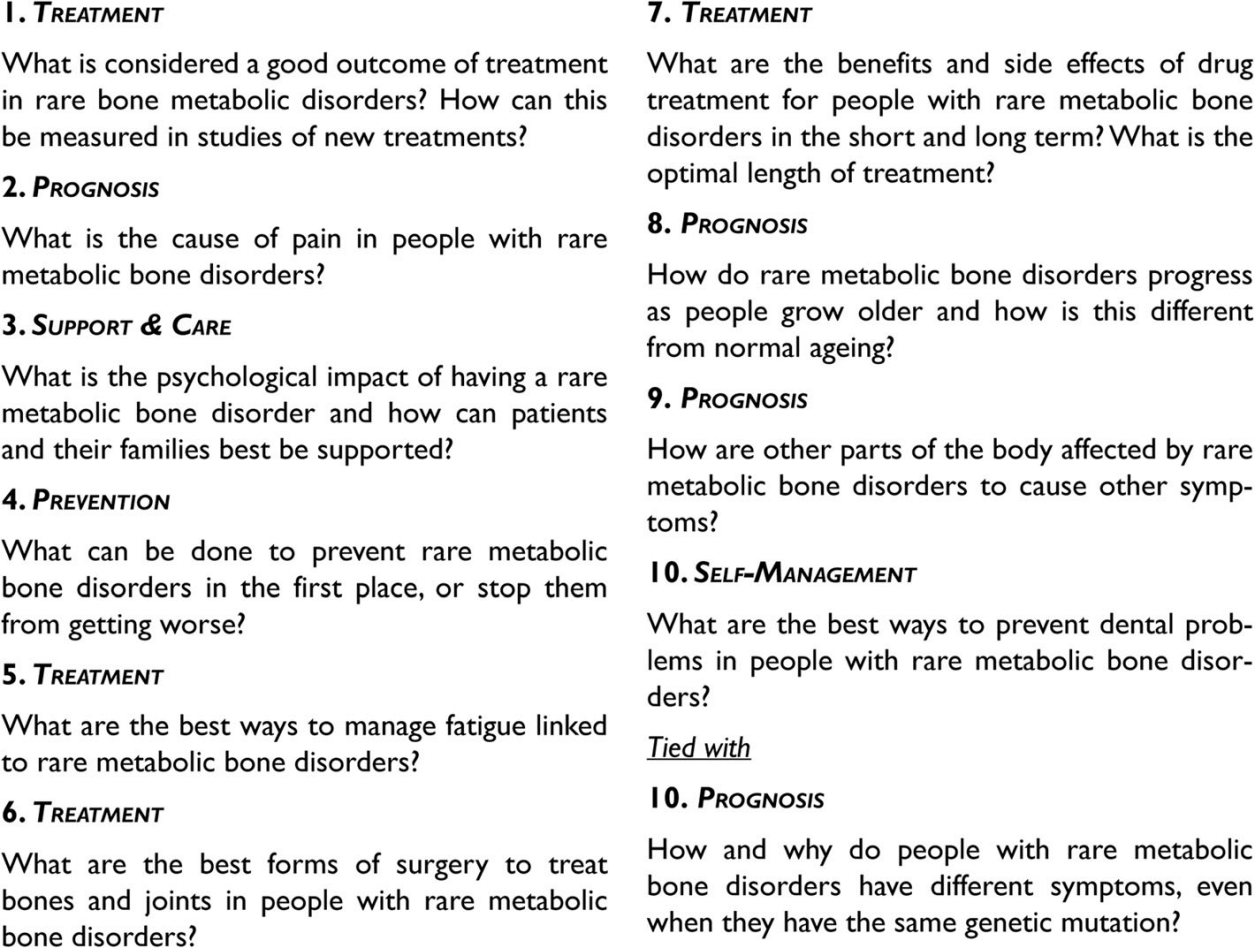
The top 10 research priorities of the rare musculoskeletal diseases in adults PSP

In the priority setting workshop, patients, carers and healthcare professionals were able to discuss the shortlist and reach a consensus on the final top 10 research priorities, which are shown in Fig. 2. All questions received from the survey of question collection, including those classified as irrelevant to this PSP, as well as the full longlist and shortlist are available on the rare musculoskeletal diseases in adults PSP website [18].

## Discussion

Following the established JLA methodology, this priority setting partnership identified the top 10 research uncertainties for three rare metabolic bone disorders – OI, XLD and FD/MAS. This was achieved with close and continuous patient, carer and healthcare professional involvement, ensuring research priority relevance for improving disease treatment and long-term management. The final 10 priorities addressed aspects of treatment, natural disease history (prognosis), self-management, support and care as well as prevention. The main overarching focus of these questions appears to be the need for better understanding of rare metabolic bone disease progression in adulthood and what treatments and management approaches provide most effective, long-term symptom management.

This PSP is the first to investigate any metabolic bone disorder. Furthermore, it addressed rare disease, which out of over a hundred completed official and JLA-associated partnerships thus far only few PSPs had done before [19–22]. Previous scientific literature on XLD, OI and FD/MAS has highlighted several of the questions identified as the top 10 research priorities within this project, namely the lack of scientific research on appropriate treatment outcomes, surgical interventions, dental problem prevention, as well as benefits, side effects, dosing and length of pharmacological therapies [1, 3, 23–25]. Furthermore, previous publications have identified the increasing importance of addressing disease management aspects applicable to adults, as more patients progress into adulthood due to improved childhood therapies [3]. However, this work, to our knowledge, is the first in the field to thoroughly describe the research demand for better rare metabolic bone disease management in adults and highlight aspects important to patients that otherwise could have been overlooked by research.

Of special note are the two “unrecognised knowns” identified during this PSP, i.e. uncertainties expressed by survey respondents that in fact can be answered confidently by the existing research evidence. Whilst the question on the optimal imaging technique was asked only once, variations of the question about the diagnosis were submitted on 10 separate occasions [18]. Even so, as these questions were not included in the prioritisation process, it remains unclear how relevant they are to the patient, carer and healthcare professional groups overall. Their presence might indicate an area of improvement in the effective communication of knowledge to healthcare professionals, patients and their carers.

Several aspects ensured the relevance and impact of this PSP to the future research on rare metabolic bone disorders. The adopted JLA approach, continued guidance by the JLA adviser and involvement of all relevant stakeholder groups assured adherence to an established, rigorous and transparent process. Open access of the full list of questions with unchanged original respondent submissions upholds these qualities in the result publication stage as well [18]. This also allows stakeholders to investigate other research priorities that did not reach the top 10. Furthermore, even though this PSP focused on rare disorders, adequate patient and carer involvement was achieved through the efficient use of the steering group members’ contacts and through collaboration with the previously mentioned patient organisations and charities, allowing the PSP to reach participants from both the UK and outside. In addition, we anticipate that the strategy of investigating several related rare diseases within a single PSP eased the recruitment of a larger sample of participants. Finally, this PSP provided research priorities that are formulated in a deliberately broad fashion. It was previously reported that broader treatment uncertainties are ranked higher in prioritisation exercises [16]. We expect that such is the case because broader questions are applicable to more people and, within this project, conditions, making the potential answers more impactful for the overall healthcare provision.

However, this work also carries a few limitations. The survey respondent group was under-representative of ethnic minorities and was mostly female. In addition, most respondents were patients and comparatively few carers and healthcare professionals responded, although this skew was partially corrected by separate prioritisation analysis in the second survey. Lastly, it is possible that the most severely affected patients were under-represented within this PSP due to their limited ability to participate, both during the survey and the final workshop stages.

In the future, we encourage researchers, funding bodies and other stakeholders to promote and use the top 10 research priorities in guiding their work. The adopted approach for this project enabled us to identify the uncertainties relevant to those who should benefit the most from the research on rare metabolic bone diseases – the patients. Hence, we hope that the findings of this PSP will minimise the research mismatch [4, 5] and maximise the impact of future research in improving therapy.

## Conclusions

This JLA PSP has identified the top 10 research priorities most relevant to adult patients with rare musculoskeletal disease, their carers and healthcare professionals. It is the first to do so for any metabolic bone disease and one of the few addressing rare disease, where little high-quality research exists, and funding is especially limited. Hence, the top 10 priorities should serve as guidelines for future research work on rare musculoskeletal disease, maximising patient benefit and reducing funding waste on projects that are less urgent.

## Data Availability

The dataset supporting the conclusions of this article is available in the NIHR JLA website: http://www.jla.nihr.ac.uk/priority-setting-partnerships/rare-musculoskeletal-diseases-in-adulthood/rare-musculoskeletal-diseases-in-adulthood.htm.

## Abbreviations

JLA: James Lind Alliance
PSP: Priority Setting Partnership
FD/MAS: Fibrous dysplasia/McCune-Albright syndrome
XLH: X-linked hypophosphatemia
OI: Osteogenesis imperfecta

## References

[1] Linglart A, Biosse-Duplan M, Briot K, Chaussain C, Esterle L, Guillaume-Czitrom S (2014). Therapeutic management of hypophosphatemic rickets from infancy to adulthood. Endocr Connect.

[2] Javaid MK, Boyce A, Appelman-Dijkstra N, Ong J, Defabianis P, Offiah A (2019). Best practice management guidelines for fibrous dysplasia/McCune-Albright syndrome: a consensus statement from the FD/MAS international consortium. Orphanet J Rare Dis.

[3] Marini JC, Forlino A, Bächinger HP, Bishop NJ, Byers PH, Paepe AD (2017). Osteogenesis imperfecta. Nat Rev Dis Primers.

[4] Tallon D, Chard J, Dieppe P (2000). Relation between agendas of the research community and the research consumer. Lancet.

[5] Liberati A (2011). Need to realign patient-oriented and commercial and academic research. Lancet.

[6] Thornton H (2008). Patient and public involvement in clinical trials. BMJ.

[7] Chalmers I, Bracken MB, Djulbegovic B, Garattini S, Grant J, Gülmezoglu AM (2014). How to increase value and reduce waste when research priorities are set. Lancet.

[8] Mockford C, Staniszewska S, Griffiths F, Herron-Marx S (2012). The impact of patient and public involvement on UK NHS health care: a systematic review. Int J Qual Health Care.

[9] Partridge N, Scadding J (2004). The James Lind Alliance: patients and clinicians should jointly identify their priorities for clinical trials. Lancet.

[10] JLA Guidebook | James Lind Alliance. Available at: www.jla.nihr.ac.uk/jla-guidebook/. Accessed 8 Jan 2020.

[11] Crowe S, Fenton M, Hall M, Cowan K, Chalmers I (2015). Patients', clinicians' and the research communities' priorities for treatment research: there is an important mismatch. Res Involv Engagem.

[12] The PSPs | James Lind Alliance. Available at: www.jla.nihr.ac.uk/priority-setting-partnerships/. Accessed 8 Jan 2020.

[13] Rangan Amar, Upadhaya Sheela, Regan Sandra, Toye Francine, Rees Jonathan L (2016). Research priorities for shoulder surgery: results of the 2015 James Lind Alliance patient and clinician priority setting partnership. BMJ Open.

[14] Davies Benjamin M., Khan Danyal Z., Mowforth Oliver D., McNair Angus G. K., Gronlund Toto, Kolias Angelos G., Tetreault Lindsay, Starkey Michelle L., Sadler Iwan, Sarewitz Ellen, Houlton Delphine, Carter Julia, Kalsi-Ryan Sukhvinder, Aarabi Bizhan, Kwon Brian K., Kurpad Shekar N., Harrop James, Wilson Jefferson R., Grossman Robert, Curt Armin, Fehlings Michael G., Kotter Mark R. N. (2019). RE-CODE DCM (REsearch Objectives and Common Data Elements for Degenerative Cervical Myelopathy): A Consensus Process to Improve Research Efficiency in DCM, Through Establishment of a Standardized Dataset for Clinical Research and the Definition of the Research Priorities. Global Spine Journal.

[15] Sheehan WJ, Williams MA, Paskins Z, Costa ML, Fernandez MA, Gould J (2019). Research priorities for the management of broken bones of the upper limb in people over 50: a UK priority setting partnership with the James Lind Alliance. BMJ Open.

[16] Fernandez MA, Arnel L, Gould J, McGibbon A, Grant R, Bell P (2018). Research priorities in fragility fractures of the lower limb and pelvis: a UK priority setting partnership with the James Lind Alliance. BMJ Open.

[17] Schoemaker CG, Armbrust W, Swart JF, Vastert SJ, van Loosdregt J, Verwoerd A (2018). Dutch juvenile idiopathic arthritis patients, carers and clinicians create a research agenda together following the James Lind Alliance method: a study protocol. Pediatr Rheumatol Online J.

[18] Rare Musculoskeletal Diseases in Adulthood | James Lind Alliance. Available at: www.jla.nihr.ac.uk/priority-setting-partnerships/rare-musculoskeletal-diseases-in-adulthood/rare-musculoskeletal-diseases-in-adulthood.htm. Accessed 10 Jan 2020.

[19] Top 10s of priorities for research | James Lind Alliance. Available at: www.jla.nihr.ac.uk/top-10-priorities/. Accessed 11 Jan 2020.

[20] Rare Inherited Anaemias | James Lind Alliance. Available at: www.jla.nihr.ac.uk/priority-setting-partnerships/rare-inherited-anaemias/. Accessed 11 Jan 2020.

[21] Davila-Seijo P, Hernández-Martín A, Morcillo-Makow E, de Lucas R, Domínguez E, Romero N (2013). Prioritization of therapy uncertainties in Dystrophic Epidermolysis Bullosa: where should research direct to? an example of priority setting partnership in very rare disorders. Orphanet J Rare Dis.

[22] Mollan S, Hemmings K, Herd CP, Denton A, Williamson S, Sinclair AJ (2019). What are the research priorities for idiopathic intracranial hypertension? A priority setting partnership between patients and healthcare professionals. BMJ Open.

[23] Thomas IH, DiMeglio LA (2016). Advances in the classification and treatment of osteogenesis imperfecta. Curr Osteoporos Rep.

[24] Robinson C, Collins MT, Boyce AM (2016). Fibrous Dysplasia/McCune-Albright Syndrome: Clinical and Translational Perspectives. Curr Osteoporos Rep.

[25] Carpenter TO, Imel EA, Holm IA, Jan de Beur SM, Insogna KL (2011). A clinician's guide to X-linked hypophosphatemia. J Bone Miner Res.

